# Endoscopic Transorbital Approach and Transcranial Approach in Spheno-Orbital Meningiomas: A Comparative Qualitative and Preliminary Quantitative Anatomical Study

**DOI:** 10.3390/jcm14196744

**Published:** 2025-09-24

**Authors:** Roberta Costanzo, Marcello D’Andrea, Roberto Manfrellotti, Jon Kristinn Nielsen, Roberto Tafuto, Alessia Tomassini, Domenico Gerardo Iacopino, Rosario Maugeri, Luigino Tosatto, Alberto Prats-Galino, Alberto Di Somma, Joaquim Enseñat

**Affiliations:** 1 Department of Neurosurgery, Villa Sofia Hospital, 90146 Palermo, Italy; robertacostanzo3@gmail.com; 2 Department of Neurosurgery, Maurizio Bufalini Hospital, 47521 Cesena, Italy; marcello.dandrea@auslromagna.it (M.D.); alessia.tomassini@auslromagna.it (A.T.); luigino.tosatto@auslromagna.it (L.T.); 3 Laboratory of Surgical Neuroanatomy, Faculty of Medicine, Universitat de Barcelona, 08035 Barcelona, Spain; manfredr@hotmail.it (R.M.); aprats@ub.edu (A.P.-G.); 4 Department of Neurosurgery, Aarhus University Hospital, 8200 Aarhus, Denmark; 5 Department of Neurosurgery, Sant’Anna and San Sebastiano Hospital, 81100 Caserta, Italy; rob.tafuto@gmail.com; 6 Unit of Neurosurgery, Post Graduate Residency Program in Neurosurgery, Department of Biomedicine, Neuroscience and Advanced Diagnostics, University of Palermo, 90127 Palermo, Italy; gerardo.iacopino@gmail.com (D.G.I.); rosario.maugeri1977@gmail.com (R.M.); 7 Department of Neurosurgery, Hospital Clinic, 08036 Barcelona, Spain; jensenat@clinic.cat

**Keywords:** spheno-orbital meningioma, skull base, endoscopy, transorbital approach, neuroanatomy, transcranial

## Abstract

**Background/Objectives**: Spheno-orbital meningiomas (SOMs) are rare tumors described as benign despite their aggressive, osteodestructive behavior. Their invasive nature makes surgical management complex and often precludes complete excision. Reaching structures within the orbit remains challenging, particularly due to insufficient exposure or overly long surgical pathways. This study aimed to provide a qualitative anatomical description of two approaches—transcranial and endoscopic transorbital—and a preliminary quantitative analysis to assess the distances required to reach the anatomical areas most commonly involved in SOMs. **Methods**: Anatomical dissections were performed on five specimens (ten sides) at the Laboratory of Surgical Neuroanatomy (LSNA), University of Barcelona. Each specimen underwent pre- and post-dissection CT scans, and all data were organized using the BrainLab^®^ Workstation System. **Results**: Both approaches provided good exposure of structures deeply involved in SOMs. A description of the superior orbital fissure (SOF), inferior orbital fissure (IOF), and optic canal (OC) was achieved from both perspectives. The preliminary quantitative analysis showed significantly shorter distances to key anatomical targets using the endoscopic transorbital approach (ETOA) compared to the transcranial route. **Conclusions**: Currently, despite significant advances, the choice of the optimal surgical approach remains debated, reflecting the complexity of balancing tumor control with functional preservation. Both approaches allow for thorough evaluation of the orbital region, offering precise anatomical insights useful for SOM management.

## 1. Introduction

Spheno-orbital meningiomas (SOMs) are rare benign tumors that arise from the meninges covering the lesser sphenoid wing and the orbital region [1,2]. They may simultaneously involve multiple anatomical structures, including intradural components and, more importantly, bone. SOMs are complex lesions whose highly invasive and osteodestructive characteristics pose significant anatomical and surgical challenges [3]. Over the years, various approaches and surgical strategies have been described; however, one of the main limitations during resection is the frequent inability to achieve complete removal (Simpson Grade I).

In some cases, invasive surgical approaches such as large craniotomies and craniofacial osteotomies have been required, resulting in considerable cosmetic morbidity (e.g., muscle atrophy, extensive scarring, bony defects) [1,2,4,5,6]. Currently, there is no unanimous consensus on the optimal surgical approach. More recently, attention has shifted toward “symptom-oriented surgery.” Accordingly, in the past decade, the scientific community has pursued the identification and refinement of surgical corridors through anatomical studies, aiming to minimize morbidity while enabling the widest possible resection with minimal postoperative complications [1,2,7].

Nevertheless, comparative anatomical studies that provide both a qualitative evaluation of the exposure achieved by these approaches and a quantitative assessment of the distances required to reach structures typically affected by hyperostotic reaction in SOMs remain scarce.

Herein, the aim of the present anatomical study is to compare two different surgical routes (ETOA and the transcranial approach) through cadaveric dissection, evaluating both the most commonly involved anatomical structures and the distances required to reach them. Although several clinical series have confirmed the safety and effectiveness of the ETOA in selected cases, and anatomical reports have explored its feasibility, direct comparative intra-specimen analyses of transcranial and transorbital routes remain limited. Our study provides both a qualitative anatomical comparison and a preliminary quantitative evaluation of access distances, thereby offering a novel perspective on the relative strengths of these approaches.

## 2. Methods

### 2.1. Qualitative and Quantitative Study

Anatomical dissections were performed on five specimens (ten sides) at the Laboratory of Surgical Neuroanatomy (LSNA) of the University of Barcelona, Spain. The two approaches were not performed sequentially on the same side. Instead, the transcranial approach was carried out on one side of each specimen and the ETOA on the contralateral side. This strategy avoided potential order effects related to sequential drilling or peeling and allowed for a direct intra-specimen comparison of the two routes. All measurements were performed independently by two neurosurgeons using the BrainLab^®^ Workstation System (Brainlab AG, Munich, Germany). The observers were blinded to the surgical approach in order to minimize bias. Each specimen underwent a pre-dissection brain CT scan, a post-single approach scan, and a final CT scan after the combined approach, with 0.5 mm thick axial spiral sections and a 0° gantry angle. The transcranial approaches were performed using a surgical microscope, while the ETOAs were performed using a rigid endoscope of 4 mm in diameter and 18 cm in length, with 0° lenses (Karl Storz, Tuttlingen, Germany). All data were uploaded to the BrainLab^®^ Workstation System, firstly for navigation guidance and point registration during dissection and, then to perform the preliminary quantitative analysis by fusing the post- and pre-dissection CT scan of each specimen. Measurements to the SOF, OC, and IOF were consistently taken to their midpoint, rather than to the medial or lateral edge. The entry point of each trajectory was defined as the external surgical access site: the skin incision in the superior eyelid for the transorbital approach, and the craniotomy margin for the transcranial approach. For each approach, we quantified three morphometric parameters: (a) exposure area, defined as the minimal cross-sectional area orthogonal to the entry–target trajectory, measured at the narrowest portion of the corridor; (b) surgical corridor volume, defined as the three-dimensional volume encompassed by the virtual corridor along the entry–target trajectory; and (c) angles of attack, defined as the angles between the entry–target line and the cranial reference planes, calculated for the SOF, IOF, and OC. All measurements were collected bilaterally and analyzed across 10 sides (5 specimens). The rationale for measuring these data lies in their potential clinical relevance: shorter distances may translate into faster surgical access, reduced operative time, and decreased manipulation of critical neurovascular structures. These factors could lead to lower surgical morbidity and improved functional outcomes for the patient.

### 2.2. Statistical Analysis

All anatomical distance measurements were performed using BrainLab^®^ Neuronavigation Software, Version 3.0, which allowed for precise point-to-point measurement between the surgical entry point and the targeted anatomical structures (SOF, IOF, and OC). Anatomical landmarks were first registered using pre-dissection CT scans, and post-dissection scans were subsequently fused to enable accurate intraoperative distance calculations.

To standardize the methodology across specimens, the same set of anatomical landmarks was used for all measurements, and the trajectory was defined as the shortest navigable linear path from the external surgical access point to each target. Measurements were independently obtained by two experienced neurosurgeons in a blinded fashion, using the same workstation and navigation platform. For each structure (SOF, IOF, and OC), we calculated the mean value and standard deviation (mean ± SD) across the five specimens. A paired two-tailed t-test was performed to compare the mean distances between the ETOA and transcranial approaches for each anatomical target, using the same side of the specimen as the pairing factor. This statistical approach was chosen to account for intra-specimen variability. The assumption of normal distribution was tested using the Shapiro–Wilk test. Statistical significance was defined as *p* < 0.05. All analyses were conducted using SPSS software version 27 (IBM Corp., Armonk, NY, USA).

### 2.3. Endoscopic Transorbital Approach

The patient is placed in a supine position with the head in a neutral position, secured with a three-pin Mayfield-Keiss head holder (Integra LifeScience Princeton, Plainsboro, NJ, USA), and moderately rotated 5–10° to the contralateral side so that the lateral orbital wall is aligned parallel to the approach direction [8,9,10]. After local anesthesia, an incision is made along a superior eyelid wrinkle. The palpebral portion of the orbicularis muscle is then incised precisely where it joins the pre-septal and tarsal orbicularis. This incision is performed in a supero-lateral direction, preserving muscle fibers until reaching the frontozygomatic suture, taking care not to violate the “white plane” (upper tarsus, orbital septum, and levator palpebrae tendon). The periosteum is incised directly over the lateral orbital wall and then dissected. The meticulous closure of this layer at the end of the procedure is crucial for reconstruction. After exposing the lateral orbital wall, the drilling of hyperostotic bone can be accomplished [11,12].

## 3. Results

### 3.1. Qualitative Study

Anatomical dissections provide a comprehensive view of the superior orbital fissure (SOF), inferior orbital fissure (IOF), meningo-orbital band (MOB), optic canal, anterior clinoid process (ACP), and the intraorbital and intradural portions of the cranial nerves III (oculomotor), IV (trochlear), V (trigeminal), and VI (abducens).

Each dissection was performed first extradurally and then intradurally, with a focus on the anatomical structures most involved and infiltrated in SOMs. To simplify the results obtained, we divided the dissection into the transcranial approach and the ETOA.

- Transcranial approach

**Figure 1:**
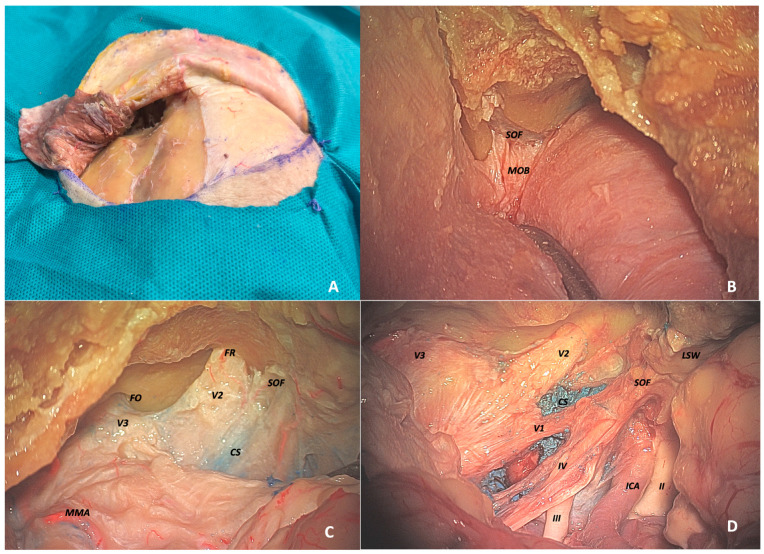
Anatomic dissection via left transcranial approach. (**A**) Interfascial dissection of the temporal muscle. (**B**) Extradural exposure of the meningo-orbital band with dural peeling over the superior orbital fissure. MOB (meningo-orbital band). (**C**) Extradural view showing MMA (middle meningeal artery), FO (foramen ovale), FR (foramen rotundum), SOF (superior orbital fissure), and CS (cavernous sinus). (**D**) Intradural exposure: from left to right—V3, V2, V1, cranial nerves IV and III, ICA (internal carotid artery), LSW (lesser sphenoid wing), SOF, and cranial nerve II.

In the case of a transcranial approach, reaching the anatomical targets occurred later in the procedure. After performing the craniotomy, extradural drilling of the greater sphenoid wing proceeded until reaching the roof of the SOF in an anteromedial position. However, before identifying it, further drilling of the lesser wing of the sphenoid was mandatory until the MOB was exposed and subsequently opened, revealing the ACP (Figure 1A,B).

At this point, the procedure could continue either extradurally or intradurally. The SOF and the OC were visible only after the complete removal of the anterior clinoid process, with the former positioned postero-anteriorly in a medio-lateral direction and the latter postero-anteriorly in a latero-medial direction (Figure 1C,D) The IOF was very difficult to identify through this approach, as reaching it would require an excessively extensive and invasive procedure.

- ETOA

**Figure 2:**
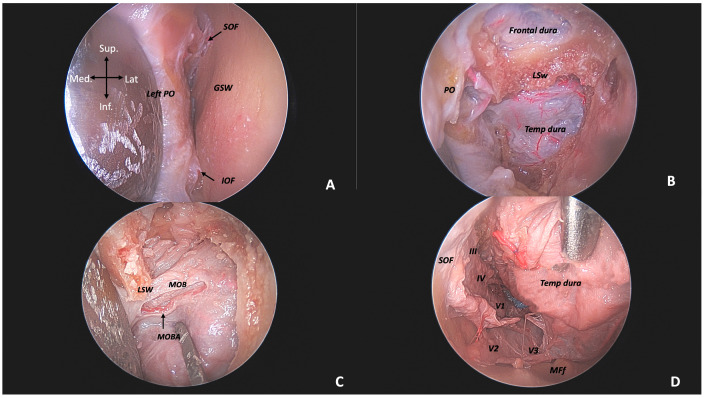
Anatomic dissection via left endoscopic transorbital approach. (**A**) End of skin phase with identification of IOF (inferior orbital fissure), SOF (superior orbital fissure), PO (periorbita), and GSW (greater sphenoid wing). (**B**) Extradural exposure of frontal and temporal dura following sagittal crest (SC) drilling. (**C**) Exposure of the meningo-orbital band (MOB) and its artery (MOBA). (**D**) Intradural view: from left to right—SOF (superior orbital fissure) with cranial nerves III, IV, and V1, FR with V2, FO with V3, and MFf (middle fossa floor).

Once the “skin phase” was completed (Figure 2A), during the second phase of the transorbital approach, the first structures that should be identified inferomedially—after opening the periosteum–periorbita layer—was the IOF, a key element in defining the bony drilling limits of the lateral orbital wall. Further retraction of the orbit allowed the identification of the SOF supero-medially, although it became visible only after exposing and drilling the sagittal crest and identifying and opening the meningo-orbital band (MOB). The opening of the MOB enabled the identification of the anterior clinoid process. (Figure 2B,C).

Drilling proceeded in a horizontal direction through the medial portion of the SOF roof, first separating it medially from the OC and then laterally from the “optic strut.” Subsequently, the dura was detached from the ACP, allowing for its removal.

Once the clinoid process was removed, the optic nerve and the SOF became visible from an extradural perspective, providing an excellent view of the III, IV, and V1 cranial nerves. The next phase involved the interdural peeling of the cavernous sinus, aiming to separate the temporal dura from the dural layer of the lateral wall of the cavernous sinus. Dissection proceeded parallel to V2 to avoid damaging the intracavernous carotid artery and the cavernous sinus itself. (Figure 2D).

**Figure 3:**
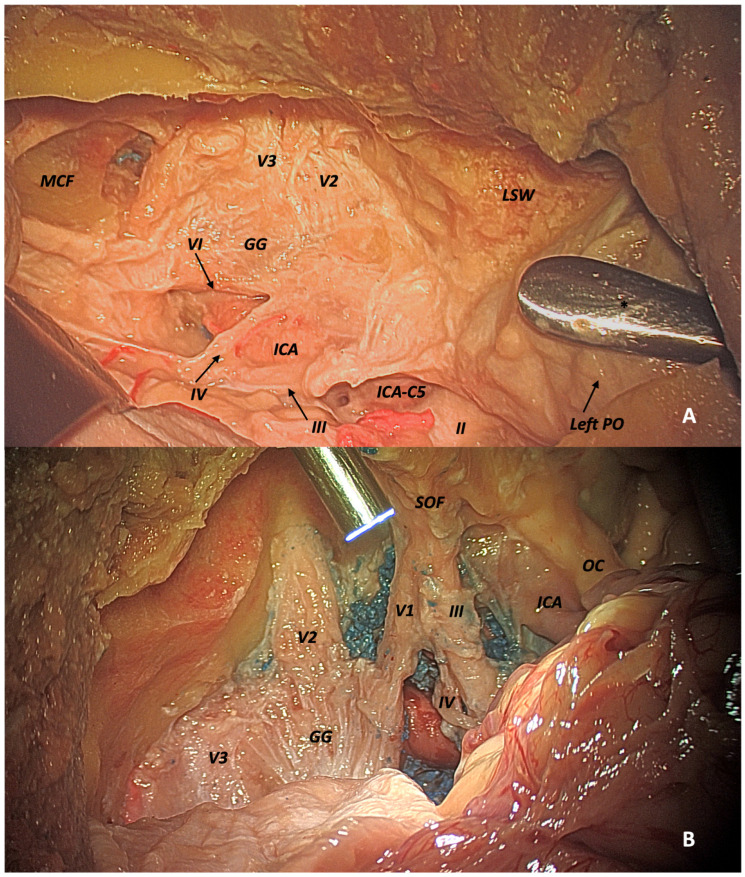
Combined transcranial and transorbital views. (**A**) Transcranial exposure showing MCF (middle cranial fossa), Gasserian ganglion (GG), ICA, lesser sphenoid wing (LSW), and (PO) periorbita. * The dissector is inserted through the transorbital approach and is used to demonstrate the extent and perspective of a combined approach. (**B**) Endoscope inserted via transorbital route, seen from transcranial view: from left to right—V3, V1, GG, (cavernous sinus) CS with intracavernous ICA, SOF with cranial nerves V1, III, IV, ICA, and OC (optic chiasm).

The following images illustrate the results of a combined approach in terms of working space and ease of reaching specific anatomical structures. (Figure 3A,B).

### 3.2. Preliminary Quantitative Study

**Figure 4:**
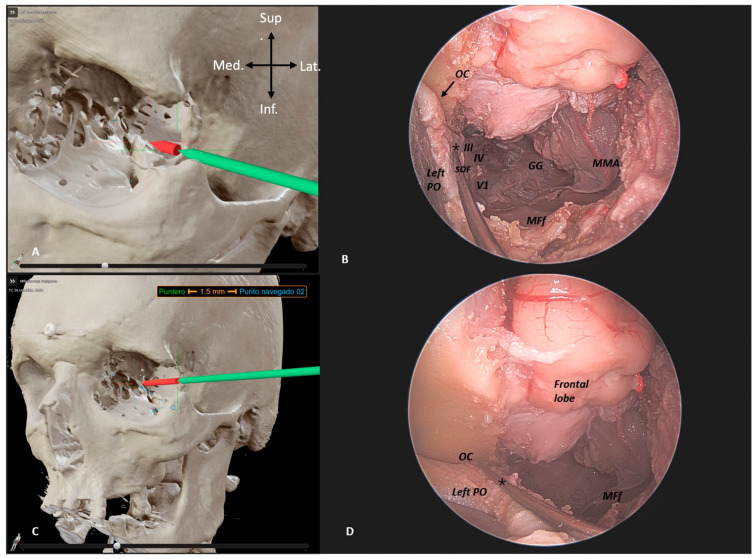
BrainLab^®^ CT reconstruction post left ETOA dissection. (**A**–**C**) Neuronavigation-guided distance measurement to key targets in spheno-orbital meningiomas. (**B**) Superior orbital fissure (SOF, *) with cranial nerves III, IV, V1; Foramen Rotondum (FR) with V2; Gasserian gangion (GG), middle meningeal artery (MMA), and middle fossa floor (MFf). (**D**) PO, optic chiasm (OC, *), frontal lobe, and MFf. * the pointer identifies the anatomical structure seen in the BrainLab^®^ CT reconstruction.

**Figure 5:**
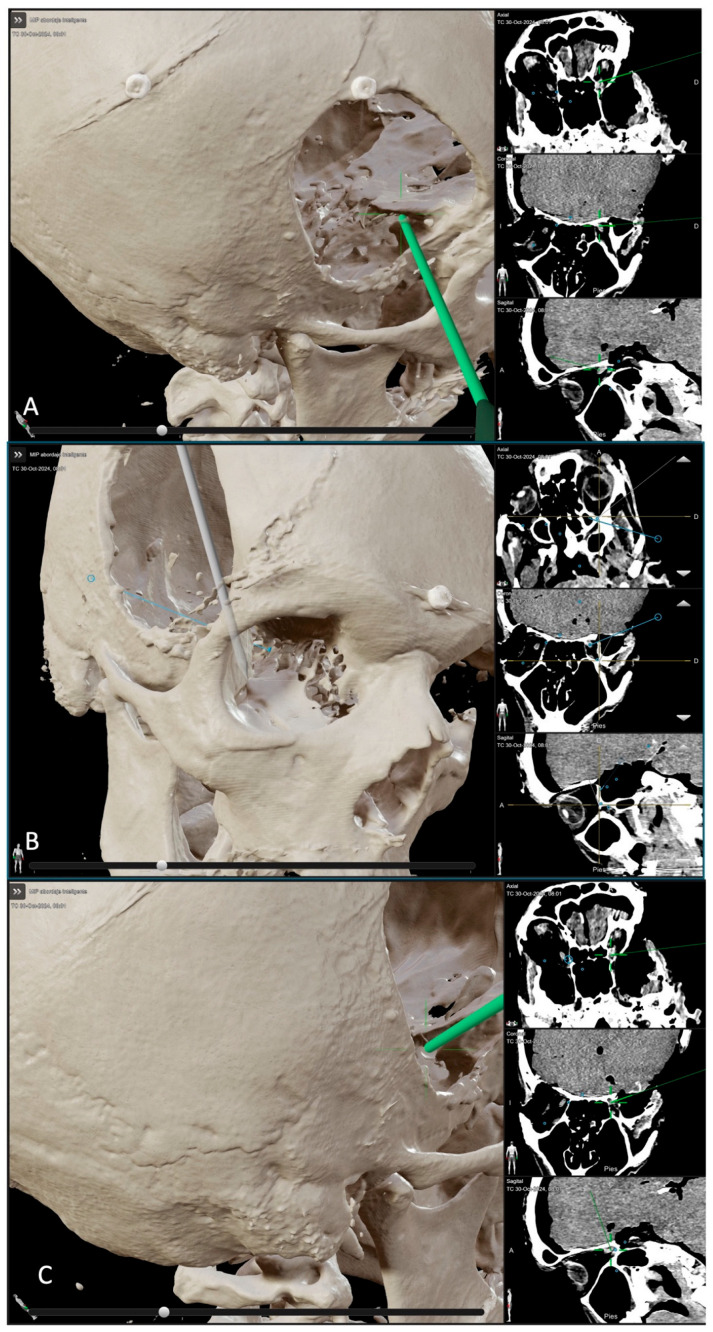
BrainLab^®^ CT reconstruction post right transcranial dissection. (**A**–**C**) Neuronavigation showing distances to SOF (**A**), IOF (**B**), and OC (**C**), relative to spheno-orbital meningiomas.

A preliminary quantitative study was also conducted to investigate the distance required to reach the SOF, IOF, and OC in both approaches. Each side of the specimen underwent both a transcranial approach and an ETOA. Afterward, the post-dissection CT scan was uploaded and used for navigation guidance and point registration during dissection. (Figure 4 and Figure 5).

The rationale for measuring these distances lies in their potential clinical relevance: shorter distances may translate into faster surgical access, reduced operative time, and decreased manipulation of critical neurovascular structures, potentially lowering surgical morbidity. Inter-observer reliability was assessed using intraclass correlation coefficients (ICCs), which demonstrated excellent agreement for all anatomical targets (ICC > 0.85). In the case of a discrepancy greater than 2 mm between observers, the measurement was repeated, and consensus was reached through discussion.

BrainLab^®^ Neuronavigation Software was used during the whole procedure to calculate the main distance to reach the abovementioned anatomical regions. All the measures obtained were finally verified using the BrainLab^®^ workstation. We obtained 30 measures from five specimens defining the ETOA as the shorter and faster way to reach these structures, often infiltrated by SOMs. Our results suggested that the shorter route was represented by the ETOA, especially to reach the IOF (72 vs. 24.4 mm) and the SOF (61.4 vs. 36.2 mm). All comparisons yielded statistically significant differences (*p* < 0.001), with the ETOA consistently demonstrating shorter distances to the target structures.

The following table shows in detail the results obtained (Table 1).

**Figure 6:**
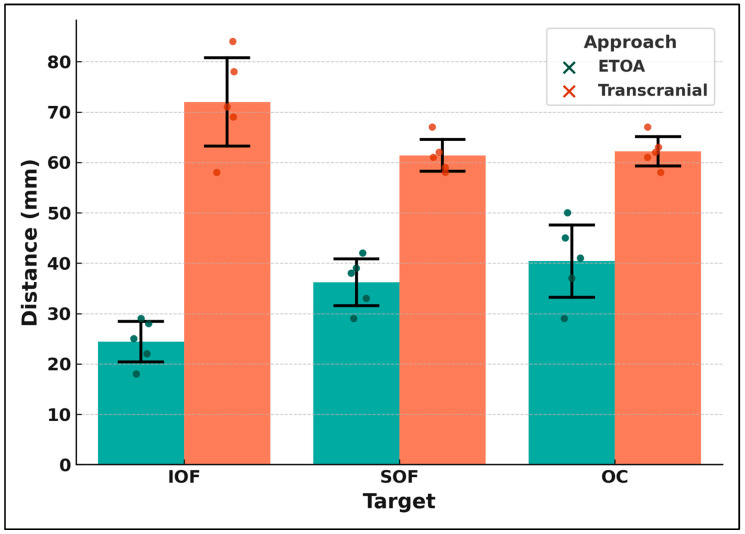
Distances to anatomical targets (IOF, SOF, OC) comparing ETOA and transcranial approaches. Bars represent mean ± SD, with individual specimen data points (dot plots).

To strengthen these findings, both paired t-tests and Wilcoxon signed-rank tests were performed, yielding concordant results. Mean differences with 95% confidence intervals and effect sizes are reported in Table 2, while individual specimen data are shown in Table 2 and Figure 6. Quantitative analysis confirmed significant differences between the two approaches. The mean exposure area was markedly smaller for the ETOA compared to the transcranial approach (≈460 mm^2^ vs. ≈1500 mm^2^, *p* < 0.01). Corridor volumes were consistently lower for the ETOA (≈2600 mm^3^) compared with transcranial corridors (≈15,900 mm^3^, *p* < 0.01). Angles of attack also differed substantially: the ETOA provided narrower but more direct corridors toward the targets (SOF: ~20–27°, IOF: ~24–30°, OC: ~30–34°), whereas the transcranial approach was associated with much wider but more oblique angles (SOF: ~78–95°, OC: ~79–90°); the IOF was not directly accessible transcranially. These findings support the complementary profiles of the two approaches, with the ETOA offering a focused and direct route, while the transcranial approach provides a wider but less direct exposure (Table 3). The significantly shorter distances observed in the ETOA for accessing key anatomical structures such as the SOF, IOF, and OC suggest a more direct and potentially less traumatic surgical route. This has notable implications:

- *SOF:* Closer access via the ETOA may reduce manipulation of cranial nerves III, IV, and V1, which traverse this region, potentially decreasing the risk of postoperative oculomotor deficits.
- *OC:* The reduced working distance may allow for more controlled extradural clinoidectomy and optic canal decompression. This is particularly relevant in SOMs involving the OC, where manipulation of the canalicular segment of the optic nerve is associated with visual outcome.
- *IOF:* The ETOA provides a more direct inferomedial trajectory, making it advantageous for the early identification and decompression of this structure, which may improve intraorbital decompression and reduce proptosis.

These anatomical advantages may also improve vascular control of the clinoidal segment of the internal carotid artery (ICA), especially during extradural dissection and cavernous sinus peeling. However, while shorter distances may facilitate surgical access, they do not automatically imply better clinical outcomes. Therefore, caution should be exercised in generalizing these anatomical findings, and future studies should correlate these data with clinical endpoints such as extent of resection and symptomatic relief.

## 4. Discussion

Although SOMs are considered a rare entity among intracranial meningiomas, they represent a true neurosurgical challenge today. Due to the extreme invasiveness of these lesions, achieving a Simpson I resection is highly complex, particularly when hyperostosis is extensive and involves structures that are difficult to access using standard transcranial approaches [1,13,14]. Our anatomical study adds to this context by providing concrete quantitative data that clarify how certain access routes—specifically the ETOA—offer significantly shorter distances to key anatomical structures often infiltrated by SOMs. This difference may have a direct impact on the surgical efficiency and safety of decompression procedures.

However, according to Chotai and Schwartz, the very definition of maximal resection should be reconsidered, as it may be misleading: applying the Simpson grading system to SOMs can inevitably lead to misinterpretation of postoperative data. Instead, calculating residual volume using postoperative MRI is more accurate and reliable [15].

Another issue widely discussed over the past decade is the choice of the best approach to maximize resection while simultaneously preserving quality of life. A 2021 meta-analysis examining 1486 patients who underwent SOM resection via transcranial approaches concluded that these approaches are effective and safe for treating these tumors [16]. Indeed, for years, the pterional approach and its variants have been considered the gold standard. However, it is important to acknowledge the risks associated with extensive osteotomies and/or craniotomies, which can be highly invasive and lead to large surgical scars, bone defects, temporal muscle atrophy, and both aesthetic and functional alterations [16,17,18,19,20,21,22].

While both the transcranial and ETOA have strengths, it is crucial to recognize their limitations. The transcranial approach may offer broader access but carries a higher risk of invasiveness and morbidity. Conversely, the ETOA, although minimally invasive, may not be suitable for tumors with significant intradural or posterior extension.

Careful preoperative evaluation remains pivotal to selecting the appropriate approach based on both tumor extension and the anatomical structures primarily involved. For instance, if the OC or the SOF are infiltrated and symptomatic, then the shorter, more direct route via the ETOA may allow for earlier and safer decompression. On the contrary, transcranial approaches remain superior when facing extensive intradural involvement, vascular encasement, or deep posterior extension. In this scenario, where finding the “right compromise” is challenging, the focus of surgery has shifted toward being more “symptoms-oriented”—aimed at treating symptoms rather than achieving gross total resection, which is often not feasible. Over the years, alternative, less invasive approaches such as the transorbital route have been explored to specifically target symptoms. Preliminary literature data, indeed, have demonstrated that elderly patients with small lesions can benefit significantly from this approach, particularly in terms of reduced hospital stay and lower surgical invasiveness [23].

The choice of approach, however, depends on two key factors: the surgeon’s expertise and the extent of the lesion. A lesion extending into the infratemporal fossa or one with excessively large dimensions would undoubtedly benefit more from a transcranial approach, as clearly illustrated in the abovementioned case.

The preliminary quantitative analysis performed in our study revealed that the ETOA provides significantly shorter access distances to all key anatomical structures evaluated. The IOF, often contributing to orbital volume and proptosis, was reached in 24.4 mm via the ETOA versus 72 mm transcranially. Similarly, the SOF (36.2 mm vs. 61.4 mm) and OC showed shorter paths with the ETOA. These differences may reduce surgical time and manipulation of neurovascular elements, particularly beneficial in decompression-focused, symptom-oriented procedures. Moreover, the smaller exposure area and surgical corridor volume of the ETOA correspond to a more focused and less invasive bone removal, potentially translating into reduced surgical morbidity and faster recovery in selected patients. In contrast, the larger exposure and volumes of the transcranial route provide greater maneuverability and multi-compartment access, but at the cost of wider exposure and tissue disruption. These quantitative differences highlight the complementary nature of the two approaches and can help guide surgical strategy according to whether precision or broader exposure is required.

Indeed, the anatomical targets analyzed in this study are directly linked to clinically relevant outcomes: OC involvement is associated with visual acuity and field impairment, SOF pathology affects oculomotor function, and IOF or diffuse orbital hyperostosis typically presents with proptosis. In this context, early decompression of the OC and SOF via the ETOA may be particularly valuable in patients experiencing progressive visual decline or cranial nerve dysfunction, as this approach provides a shorter and more direct surgical pathway to these structures. Placed within current indication and training frameworks, our observed reductions in access distance—approximately 48 mm to the IOF, 25 mm to the SOF, and 22 mm to the OC—are most likely to be decisive when the surgical objective is focused on decompression of these sites in symptom-oriented cases and in elderly or comorbid patients with limited intradural/posterior extension. Conversely, transcranial approaches remain preferable in the presence of extensive intradural involvement, posterior extension, or vascular encasement, where broader exposure and reconstruction are required.

Symptom-based outcomes such as visual acuity, oculomotor function, and proptosis may therefore benefit from the more direct and less traumatic anatomical access that the ETOA offers to the SOF and OC.

- Outcome

Negative predictive factors that can influence clinical outcomes include OC infiltration (leading to proptosis, visual acuity deficits, and visual field defects), periorbital involvement (visual acuity), and intracranial extension (visual field deficits). Additionally, the duration of preoperative symptoms is a strong predictor of postoperative recovery [16,23]. Scientific evidence has documented long-term improvements in both visual acuity and visual field in patients undergoing either approach [16,18].

Proptosis is one of the most frequent clinical signs in SOMs, and it can result from a hyperostotic reaction, intraorbital tumor growth, or venous obstruction due to SOF infiltration. This underlines the importance of SOF decompression in surgical planning. Given the ETOA’s more favorable angle of access and shorter trajectory to this region, it may offer a more efficient and focused approach in selected cases.

Long-term outcomes of transcranial approaches have shown a significant reduction in proptosis. However, for the ETOA, additional studies are needed to confirm its effectiveness, as it remains a relatively new approach with limited follow-up data. The same observation applies to visual outcomes: available case studies are still limited, but preliminary results appear promising [23,24,25,26]. Conversely, transcranial approaches, with their longer follow-up data, have documented cases of worsening proptosis due to rapid tumor regrowth, scarring, or subtotal resection [27].

Another challenge is the current lack of long-term follow-up data, particularly for patients treated with the ETOA. To fully validate its safety, efficacy, and recurrence rates, structured long-term clinical studies are essential. These should assess not only radiographic results but also functional status, visual outcomes, and quality of life.

- Reconstruction techniques

Regarding reconstruction techniques, in ETOA cases, reconstructing the orbital rim is necessary, while reconstruction of the lateral orbital wall remains debated. In many cases, the lateral wall is not reconstructed to allow natural proptosis correction and achieve better aesthetic results. Proper reconstruction aims to minimize the risk of enophthalmos, CSF fistula, and diplopia, while improving the cosmetic outcome of the frontotemporal region [23,28].

In transcranial approaches, various reconstruction techniques are employed to achieve an optimal aesthetic and functional outcome, ensuring proper repositioning of the eyeball. Some studies have proposed the use of autologous fat, as rigid reconstruction may not always be beneficial—creating a “soft space” can facilitate proptosis resolution while preventing enophthalmos [17,21,29,30]. However, the combination of fat and rigid prosthesis has not proven effective due to the high risk of “overpacking” and ocular dysfunction [13,31,32]. This observation, although supported by anecdotal evidence and limited case reports, would benefit from additional clinical data or comparative studies to confirm its generalizability.

On the other hand, the use of 3D printing technology, as presented in the illustrative transcranial case, represents a significant advancement in SOM treatment. With computer-aided design (CAD) and manufacturing (CAM), preoperative calculation of the bone volume to be removed and the volume necessary for optimal eye repositioning has led to excellent aesthetic outcomes [6,17]. Herein, in the following table, we illustrated a summary of the reconstruction strategies for each approach. (Table 4).

- Which one is the best approach?

The choice of approach depends on multiple factors. According to the recent classification of transorbital approach difficulty levels (I–V), the first scenario involves a purely extradural hyperostotic lesion (Grade I), while the second involves a hyperostotic lesion with an associated intradural component (Grade II). Based on this classification, the ETOA has a well-defined indication for treating SOMs, while also serving as a model case for learning the technique. Training and translational anatomy play key roles in the development of young neurosurgeons and in refining the learning curve, significantly reducing training time [33,34,35].

Transcranial approaches allow good exposure of the lateral orbital wall, the orbital roof, and the intradural component. In contrast, the ETOA enables lateral orbital wall removal, periorbital exploration, and drilling of the hyperostotic component, ensuring excellent SOF decompression. When a large intradural component is present, a transcranial approach is preferable for better tumor control. The ETOA, however, is a valuable alternative for patients previously treated via a transcranial approach, those with a low Karnofsky performance status, patients over 75 years old, and those with comorbidities, as they may benefit from reduced hospitalization time [36,37,38].

Therefore, the best approach does not exist in absolute terms: it should be customized based on each patient’s individual characteristics and, most importantly, on the surgical goal—whether symptom resolution or a highly demolitive resection. Ultimately, our anatomical findings support the evolving paradigm of customized, anatomy-based surgical strategies for SOMs. By aligning the choice of approach with both the lesion’s anatomical footprint and the patient’s clinical profile, surgical teams may optimize outcomes while minimizing invasiveness.

From an anatomical perspective, different patterns of involvement may guide surgical decision-making: isolated hyperostosis may be more amenable to targeted endoscopic transorbital removal; lesions with SOF or OC involvement often benefit from the shorter, direct ETOA trajectory for decompression, while cases with major intradural extension remain better suited to a transcranial route. Although a full algorithm requires correlation with clinical outcomes, these preliminary observations may provide useful orientation for the decision-making process. While both approaches provide relevant anatomical and clinical advantages, each also carries specific risks. The ETOA requires careful orbital and globe protection to prevent ocular injury, and transient eyelid morbidity may occur; interdural peeling around the cavernous sinus must be approached cautiously to avoid neurovascular damage. Conversely, transcranial approaches are associated with temporal muscle atrophy, scarring, and cosmetic alterations. These risks can be mitigated through meticulous soft-tissue handling, appropriate reconstruction strategies, and careful preoperative planning tailored to patient-specific anatomy [2].

## 5. Limitations and Future Directions

A significant limitation of this study is that it was conducted on cadaveric specimens. While anatomical dissections offer a precise understanding of complex surgical corridors and spatial relationships, they lack the dynamic features of live surgery—such as bleeding, tissue elasticity, and physiological responses—that can influence surgical decision-making and outcomes. In particular, bleeding control, tissue characteristics, and orbital compliance cannot be reproduced in the cadaveric setting, which may affect the applicability of the anatomical measurements to real intraoperative conditions. Another essential limitation of this study is that the specimens did not present spheno-orbital meningiomas, which are frequently associated with hyperostosis. Consequently, the measured exposure areas may be underestimated compared to real surgical conditions.

Moreover, the transcranial and transorbital approaches were performed on contralateral sides rather than sequentially on the same side, so order effects related to prior drilling or peeling were inherently avoided. Nevertheless, side-to-side anatomical variability could represent a residual source of bias, and this should be considered when interpreting the quantitative results.

To mitigate this limitation, future studies should aim to correlate anatomical findings with clinical data from actual surgical cases. Integrating intraoperative observations, postoperative imaging, and patient outcomes with cadaveric measurements would significantly enhance the translational relevance of anatomical research. In this regard, a prospective study design is planned to correlate corridor metrics with operative time, extent of bony decompression achieved, and functional outcomes such as visual acuity, oculomotor function, and proptosis reduction. This approach would help validate the anatomical assumptions in real-world conditions and guide the refinement of surgical strategies.

Furthermore, although the ETOA demonstrated favorable anatomical access in our study, the relatively limited clinical follow-up available for this technique constrains our ability to draw definitive conclusions regarding its long-term efficacy and safety. Prospective future studies with standardized, long-term follow-up are essential to evaluate outcomes such as recurrence rates, visual recovery, complication rates, and patient-reported quality of life.

## 6. Conclusions

Spheno-orbital meningiomas pose a significant challenge in neurosurgery. Their aggressive and infiltrative characteristics make a total resection difficult, redefining the concept of “onco-functional balance.” However, as surgical techniques continue to evolve, surgeons are increasingly able to customize their approach to the unique characteristics of each patient. In this complex scenario, the present study provides both qualitative and quantitative analysis of two surgical strategies, which, despite differing objectives, show comparable outcomes.

Further studies are crucial to confirm the role and effectiveness of the ETOA in the treatment of these tumors. However, preliminary results are undoubtedly promising.

## Figures and Tables

**Table 1:** Quantitative analysis of distances to IOF, SOF, and OC on both sides across five specimens after transcranial and endoscopic transorbital approaches. IOF, inferior orbital fissure; SOF, superior orbital fissure; OC, optic canal; ETOA, endoscopic transorbital approach.

Specimen	Anatomical Structure	ETOA (Distance in mm)	Transcranial Approach (Distance in mm)
**1**	IOF	18	71
	SOF	41	61
	OC	45	63
**2**	IOF	34	87
	SOF	42	61
	OC	43	65
**3**	IOF	22	69
	SOF	30	63
	OC	37	61
**4**	IOF	23	55
	SOF	29	58
	OC	36	60
**5**	IOF	25	78
	SOF	39	64
	OC	41	62
			
**Mean+/** **−** **SD**	IOF	24.4 ± 5.94	72 ± 11.83
**Mean+/** **−** **SD**	SOF	36.2 ± 6.22	61.4 ± 2.30
**Mean+/** **−** **SD**	OC	40.4 ± 3.85	62.2 ± 1.92
***p*** **-value**		<0.001	

**Table 2:** Mean distances (mm) to IOF, SOF, and OC via ETOA and transcranial approaches. Values are mean ± SD (n = 5). Mean differences, 95% CI, Wilcoxon test, and effect sizes are reported.

Anatomical Structure	ETOA (Mean ± SD)	Transcranial (Mean ± SD)	Mean Difference [95% CI]	Wilcoxon (*p*)	Effect Size (Cohen’s dz)	n
**IOF**	24.4 ± 5.9	72.0 ± 11.8	−47.6 [−58.9, −36.3]	0.062	−5.23	5
**SOF**	36.2 ± 6.2	61.4 ± 2.3	−25.2 [−32.6, −17.8]	0.062	−4.25	5
**Optic Canal**	40.4 ± 3.8	62.2 ± 1.9	−21.8 [−24.9, −18.7]	0.062	−8.76	5

**Table 3:** Quantitative comparison between ETOA and transcranial approaches in 10 sides (5 specimens). Reported values include exposure area (mm^2^), corridor volume (mm^3^), and angle of attack (°) toward SOF, IOF, and OC. “/” indicates not-reachable target.

Specimen	Side	ETOA Exposure (mm^2^)	Transcranial Exposure (mm^2^)	ETOA Volume (mm^3^)	Transcranial Volume (mm^3^)	ETOA Angle SOF (°)	Transcranial Angle SOF (°)	ETOA Angle IOF (°)	Transcranial Angle IOF (°)	ETOA Angle OC (°)	Transcranial Angle OC (°)
**1**	R	385.0	1405.0	2601.1	9448.1	26.5	78.2	30.1	/	30.3	89.6
**1**	L	483.0	1575.0	1737.6	15,857.3	20.6	94.6	24.1	/	33.6	79.2
**2**	R	452.0	1653.0	3156.3	18,148.5	23.6	76.2	24.5	/	32.7	74.2
**2**	L	361.0	1515.0	3027.2	18,148.5	20.3	91.1	25.5	/	29.4	82.3
**3**	R	564.0	1538.2	2268.5	13,591.0	21.0	93.9	28.4	/	31.3	86.9
**3**	L	531.0	1530.6	2069.8	17,256.4	24.2	85.8	28.4	/	32.1	88.4
**4**	R	438.7	1542.5	3387.5	13,188.9	20.9	96.8	29.6	/	31.5	76.8
**4**	L	451.9	1681.4	2449.2	14,947.3	17.5	73.9	24.7	/	33.7	90.0
**5**	R	456.3	1426.9	2352.9	19,195.4	23.4	86.8	29.2	/	30.7	89.7
**5**	L	434.8	1464.8	3135.6	15,144.9	27.4	101.5	24.7	/	33.2	85.1
**Mean**		455.8	1533.2	2618.6	15,492.6	22.5	87.9	26.9	/	31.9	84.2
**SD**		60.4	88.8	538.9	2923.8	3.0	9.4	2.4	/	1.5	5.8

**Table 4:** Reconstruction strategies according to surgical approach.

Approach	When to Reconstruct	Structures	Materials	Trade-Offs
**ETOA**	Rim almost always; lateral wall debated (often omitted to favor natural proptosis correction).	Orbital rim; lateral wall optional.	Autologous bone, porous polyethylene, titanium mesh; sometimes no rigid material (‘soft space’).	Omission of lateral wall may help correct proptosis and aesthetics, but risk of enophthalmos; rigid reconstruction may increase diplopia.
**Transcranial**	After large osteotomies or cranio-orbital removal, rim and lateral wall reconstruction is standard.	Orbital rim + lateral wall.	Autologous bone grafts, fat grafts (‘soft space’), titanium mesh, custom 3D-printed implants (CAD/CAM).	Rigid reconstruction restores orbital contour but may cause overpacking; fat grafts reduce risk of enophthalmos and help proptosis; 3D-printed implants ensure precise orbital repositioning but require resources.

## Data Availability

Data supporting the findings of this study are available from the corresponding author upon reasonable request.

## Abbreviations

The following abbreviations are used in this manuscript:

ETOA Endoscopic transorbital approach SOM	Spheno-orbital meningioma
OC	Optic canal
SOF	Superior orbital fissure
IOF	Inferior orbital fissure
